# The Role of Working Memory Gating in Task Switching: A Procedural Version of the Reference-Back Paradigm

**DOI:** 10.3389/fpsyg.2017.02260

**Published:** 2017-12-21

**Authors:** Yoav Kessler

**Affiliations:** Department of Psychology and Zlotowski Center for Neuroscience, Ben-Gurion University of the Negev, Beersheba, Israel

**Keywords:** working memory, gating, task switching, updating, referene-back

## Abstract

Models of working memory (WM) suggest that the contents of WM are separated from perceptual input by a gate, that enables shielding information against interference when closed, and allows for rapid updating when open. Recent work in the declarative WM domain provided evidence for this notion, demonstrating the behavioral cost of opening and closing the gate. The goal of the present work was to examine gating in procedural WM, namely in a task-switching experiment. In each trial, participants were presented with a digit and a task cue, indicating whether the required task was a parity or a magnitude decision. Critically, a colored frame around the stimulus indicated whether the task cue was relevant (attend trials), or whether it had to be ignored, and the previous task set should be applied regardless of the present cue (ignore trials). Switching between tasks, and between ignore and attend trials, was manipulated. The results of two experiments demonstrated that the cost of gate opening was eliminated in task switching trials, implying that both processes operate in parallel.

Most of our daily tasks, from making coffee to crossing the road, require us to keep information in mind, to use it for guiding future actions and to update it whenever newer information arrives. Working memory (WM) is the cognitive system that enables these abilities (Baddeley and Hitch, 1974; Miyake and Shah, 1999), and WM updating is the ability to modify the stored information upon need (Morris and Jones, 1990). Updating is an ability rather than a single process, which is carried out by removing outdated items (Oberauer, 2001; Ecker et al., 2014), by adding new items, and/or by substituting the existing information with new one (Kessler and Meiran, 2006, 2008; Ecker et al., 2010; Kessler and Oberauer, 2014, 2015).

Theoretical models of WM emphasize the conflict between maintenance and updating (Frank et al., 2001; Miller and Cohen, 2001; O'Reilly, 2006; Badre, 2012; see Fallon et al., 2017, for a recent demonstration). Specifically, WM enables to maintain relevant information in a highly accessible state, shielded from being interfered by irrelevant information. However, this shielding should be removed rapidly when needed, in order to enable updating the representations held in WM. Therefore, rapid updating must counteract maintenance. The tension between these two forces is assumed to be regulated by cognitive control, in the form of a decision process that determines which items will be updated and when. The above-mentioned theoretical models account for this control by assuming a gating system, which separates WM from the flow of information that arises from internal (thoughts) or external (perception) sources. The gate serves as a selective filter. When closed, the gate enables robust maintenance within WM, while blocking irrelevant information. In contrast, opening the gate allows WM to be updated with goal-relevant input. Hence, the gate has two “states,” open and closed. Controlling these states is critical to the optimal function of WM. It is worth noting at this point that the gate metaphor typically applies to situations in which relevant and irrelevant information is presented sequentially, rather than simultaneously. In these situations, it is possible to block irrelevant information and preserve relevant one by changing the state of the gate over time, while alternating between receiving relevant and irrelevant information. Tasks that presumably involve temporally-based gating include the AX-CPT task (Braver and Cohen, 2000; D'Ardenne et al., 2012; Kessler et al., 2017), attentional blink (Raymond et al., 1992), and complex span (Daneman and Carpenter, 1980). As will be elaborated below, the goal of the present work is to examine the role of gating in task switching, and specifically the relationship between the process of gate opening to that of switching a task-set.

As mentioned above, updating is not a unitary process. Rather, based on the gating model, I suggest that WM updating is carried out by a complex cascade of sub-processes. These include: (1) detecting a change in the environment (Rensink, 2002; Hyun et al., 2009); (2) identifying the changed information as one that requires robust maintenance. In other words, identifying it as goal-relevant; (3) opening the gate to WM; (4) removing the outdated item from WM (Oberauer, 2001; Ecker et al., 2014); (5) encoding the new information into WM; and (6) closing the gate in order to shield the updated information for future distractors. Due to the large number of processes involved, it is a challenge to identify these processes, tease them apart, understand the conditions under which they take place, and specify the relationship among them. For example, it is conceivable that some processes are not required in some cases, while others are optional and depend on the task structure or individual differences. Also, the order in which these processes take place it is still not well-understood, including whether they operate serially or in parallel.

Kessler and Oberauer (2014) demonstrated that the time required to update WM is composed of two components: switching the state of the gate (namely, opening or closing), and modifying the associations between items in WM and their context (e.g., position). Participants were presented with series of trials, each trial comprised a set of 4 letter. After a varied number of trials, they were prompted to recall the most recent set of letters. Accordingly, the participants had to update their WM with the new letter set in each trial, and were not required to remember the letters presented in earlier trials. In each trial the participants had to press a key to in order to proceed to the following trial. This keypress enabled measuring the duration of WM updating. Critically, in each trial, some of the items were repeated and some were updated, compared to the previous trial. The number and serial positions of updated and repeated letters was manipulated. Updating times were explained by a scanning and gate-switching model. According to this model, in each trial participants scan the letter set according to the reading direction (i.e., left-to-right for English letters, right-to-left for Hebrew letters; see Kessler and Oberauer, 2015). While scanning, they are encountered with new items (that need to be updated into WM), and items that are repeated from the previous trial (that do not require updating). Accordingly, moving from a repeated item to an updated one requires to open the gate to WM, while moving in the other direction requires closing the gate. Updating costs increased linearly with the total number of gate switch operations. In addition, within a sequence of updated items, updating times were proportional to the number of updated items, reflecting the added cost of constituting an association between the new item and its context.

A more direct evidence for gate switching costs was provided using the *reference-back* task (Rac-Lubashevsky and Kessler, 2016a), that was developed with the aim of disentangling the sub-processes of WM updating. The reference-back is based on the n-back task, arguably one of the most commonly used measures of WM updating (e.g., Jonides et al., 1997; Chatham et al., 2011). In the “standard” n-back task, participants are presented with a stimulus in each trial, and are required to decide whether or not it is identical to the stimulus presented n trials before. Since each presented stimulus will later serve as a reference for comparison, WM updating is required in each trial of this task (with the possible exception of 1-back, see Rac-Lubashevsky and Kessler, 2016a). This fact, along with the overall computational complexity of the task, does not permit one to extract the updating process in isolation due to the lack of baseline. Also, it is not clear which trials of the n-back task trigger gate opening and closing.

The reference-back paradigm was developed in order to overcome these shortcomings. This task is composed of two types of trials, reference and comparison, which are indicated by different colors, a red or blue frame surrounding the stimulus, respectively. In each trial, participants are required to indicate whether the presented item is the same as, or different from, the most recent item that appeared within a red frame. Accordingly, each trial in this task requires a comparison to the reference followed by a same/different decision. In addition, reference trials (indicated by a red frame) require one to update WM with the presented stimulus, because it should serve as a reference to which the following trials should be compared. Thus, reference trials require opening the gate to WM, in order to enable updating. On the other hand, comparison trials (indicated by a blue frame) do not require WM updating. Instead, these trials require one to continue maintaining the last reference stimulus in WM. Because each comparison trial is also compared to the last reference trial, the reference needs to be protected from being overwritten by changes in comparison trials. Hence, the gate to WM should be closed in these trials. Previous results using this paradigm (Rac-Lubashevsky and Kessler, 2016a,b; Rac-Lubashevsky et al., 2017) demonstrated that (a) performance in reference trials is slower than in comparison trials, supporting the additional updating process required in the former, and (b) switching between the two trial types is associated with an additional cost, reflecting the time taken to open or close the gate to WM. This cost implies that the state of gate tends to remain constant, either open or closed, until a change is required (see also Kessler et al., 2017, for a similar finding using the AX-CPT task).

The behavioral evidence for gate-switching costs that were presented above arrive from paradigms that involve declarative materials (such as letters, digits) as memoranda. Oberauer et al. (Oberauer, 2009; Oberauer et al., 2013) suggested that WM is composed of analogous structures and processes that apply to declarative and procedural information. In their framework, procedural WM is the ability to maintain, update and manipulate task-sets and task-rules. As with declarative information, procedural WM is limited in capacity, is composed of associations between items (responses) and contextual information (stimuli), and depends on a selection mechanism. While the two WM systems are analogous, they process information in parallel, suggesting that they are independent (Souza et al., 2012). Based on the distinction between procedural and declarative WM, the present study sought out to examine whether gate switching also takes place in procedural WM, and how it is related to other phenomenon observed in this sub-system.

More specifically, the present study examined whether switching between task-sets (Grange and Houghton, 2014) involves gate opening. Task switching is a core executive function (Miyake et al., 2000). In a typical task switching experiment, participants are required to alternate between different sets of S-R rules (“task-sets”) that can be applied to the same stimuli. Therefore, switching between task-sets reflects cognitive flexibility, since it requires from the participant to react differently to the same perceived input. Typically, a task-cue is presented either before or together with the stimulus. The cue indicates which of the possible task-sets is relevant in the present trial (Meiran, 1996). The most important and robust finding with this paradigm is a task-switching cost. Specifically, performance (in reaction times and accuracy) is worse for switch trials, in which the relevant task is different from the one that was relevant in the preceding trial, compared to repetition trials, in which the relevant task did not change.

The ability to apply the cued task-set to a stimulus is a prime example of goal-directed behavior, in which the required action depends on the internal representation of the desired goal. In contrast to automatic performance, in which the stimulus is directly associated with its response (i.e., in long-term memory), in goal-directed behavior the response is mediated by the on-line representation of the relevant goal, which is often selected among several options. WM plays a crucial role in maintaining the relevant task set and biasing performance according to it (Miller and Cohen, 2001; Kane and Engle, 2003). Therefore, switching the relevant task is a special case of (procedural) WM updating (see Oberauer et al., 2013).

In the present work, the possible role of gating in updating WM with task-sets is examined using a procedural version of the reference-back paradigm. This is essentially a reference-back task, in which the memoranda are tasks rather than letters. Participants were required to switch between tasks upon a cue, as commonly used in the cued task-switching paradigm. Importantly, the *relevance* of the task cue was manipulated between trials. In *attend* trials, the participants were required to perform the task indicated by the cue, as in standard task switching experiments. In *ignore* trials, however, they had to ignore the cue and continue to perform the task that was relevant in the previous trial (c.f. rule violation; Pfister et al., 2016). In other words, ignore trials required to filter out the cue information, while maintaining the previously-relevant task-set in WM. Accordingly, the *attend* and *ignore* conditions correspond to *updating* and *maintenance* modes of WM operation, respectively (c.f. Kessler and Oberauer, 2014). Critically, switching between these conditions corresponds to opening and closing the gate to WM. Specifically, switching from *ignore* to *attend* involves gate opening, and switching from *attend* to *ignore* involves gate closing (see Figure 1).

**Figure 1:**
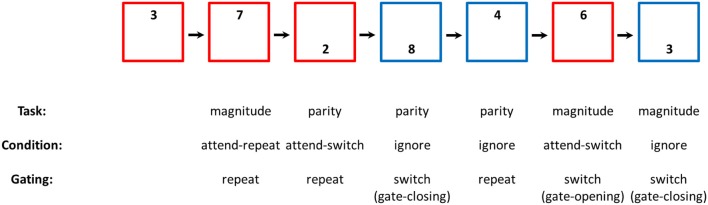
Schematic representation of Experiment 1. *Task* represent the relevant task in each trial, being a magnitude or a parity judgment. When the frame was red (*attend* trials), the participants were instructed to respond according to the position of the digit. Specifically, a magnitude judgment was required when the digit appeared at the top part of the frame, and a parity judgment was required when the digit appeared at the bottom. Attend trials that involve a task repetition are denoted *attend-repeat*, and attend trials that involve a task switch (compared to the previous trial) are denoted *attend-switch*. When the frame was blue, the participants were required to ignore the position of the digit, and continue performing the task that was relevant in the previous trial. These trials are therefore denoted *ignore* trials. Accordingly, the *Condition* in each trial was *ignore, attend-switch*, or *attend-repeat*. In addition, *Gating* indicated whether the color of the frame, which corresponds to the state of the gate to WM, was switched or repeated from the previous trials. A blue frame corresponds to a closed gate, and a red frame corresponds to an open gate. Therefore, trials in which the state of the gate was repeated from the previous trial are denoted *gate-repetition*, and trials in which the state of the gate was different from the previous trial are denoted *gate-switch*. More specifically, switching from *attend* to *ignore* trials involves *gate-closing*, and switching from *ignore* to *attend* trials involves *gate-opening*.

The procedural reference-back task enables to examine the interaction between gate opening and task switching. Specifically, *ignore* trials always involved a task-set repetition, since the cue identity was irrelevant. *Attend* trials, in contrast, could either involve a task repetition or a task switch. In addition, an *attend* trial could either follow an *ignore* trial, implying a gate opening, or follow another attend trial.

Back to the question of the relationship between task-switching and gate opening, three possible results are conceivable. One possibility is that task-switching cost is additive with gate opening cost. That is, the same task switching cost will be observed when moving from an ignore trial to an attend trial, and within a sequence of attend trials. Such a result would imply that gate opening is independent of task switching, suggesting that task switching cost does not include the duration of gate opening. A second possibility is an under-additive interaction, namely a smaller gate-opening cost in task-switch trials. This result implies a that the two processes take place in parallel. A third possibility is an over-additive interaction, reflecting a cross-talk or shared resources between the two operations.

## Experiment 1

### Method

#### Participants

Nineteen students from Ben-Gurion University of the Negev participated in the study in return for course credit or monetary compensation. All the participants were right handed, and reported having no neuropsychological deficits or learning disabilities. The study was approved by the Department of Psychology Ethics committee. All participants provided a written consent to participate in the study. One participant was removed from the analysis due to an exceptionally high error rate (27%) in one of the conditions.

#### Procedure

In each trial, one of the digits 1–9 (excluding 5) was presented at the top or the bottom of a red or a blue frame. Depending on the task, the participants had to report, using the keys “p” or “q” on a standard keyboard, whether the digit is larger/smaller than 5, or whether it is odd or even. The location of the digit within the frame served as a task cue (top = magnitude, bottom = parity). Critically, the color of the frame around the digit indicated the trial type. A red frame (*attend* trial) indicated that the task cue (namely, the location of the digit) was relevant, and the participant needed to perform the task that was associated with the cue. In contrast, a blue frame (*ignore* trial) indicated that the task cue was irrelevant, and the participant needed to continue performing the same task they performed in the previous trial, regardless of the location of the digit (see Figure 1). The trials were self-paced, separated by an ITI of 1,000 ms. The experiment included 8 blocks of 99 trials each. The first block was a practice block, and it was therefore removed from the analysis. The first trial in each block was an attend trial. The condition in each of the following trials was selected at random with equal probabilities.

### Results and discussion

Since Task-Switch is nested within Trial-Type, namely only attend trials could involve a task-switch, these variables do not create a full factorial design. I therefore created a new variable, Condition, with three levels corresponding to all possible combinations of the above variables: ignore (and hence task-repetition), attend with a task repetition, and attend with a task switch. The descriptive statistics are presented in **Appendix**. The raw data and analysis scripts are publically available through OSF, https://osf.io/x69j8.

#### RT

Error trials, as well as post-error trials, were removed from the RT analysis. Outlier removal was done in two steps. First, trials slower than 10 s were removed (0.0002% of the trials). Then, trials that deviated in more than 2 standard deviations from the mean of their condition within each subject were omitted from this analysis (4.7% of the trials).

**Figure 2:**
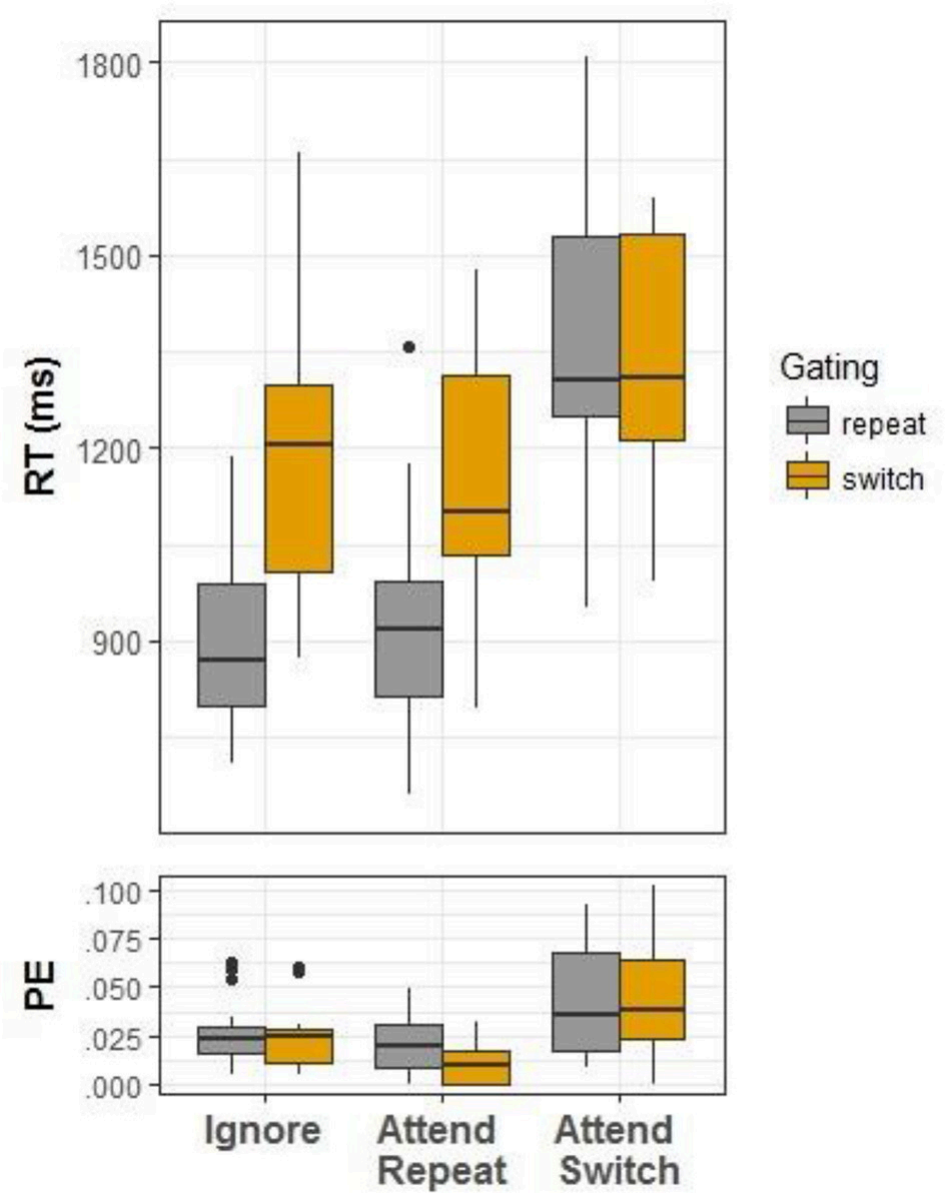
Box plots for reaction time (RT) and error proportions (PE) in Experiment 1.

An analysis of variance (ANOVA) was conducted with Condition (ignore, attend task-repetition, attend task-switch) and Gating (repeat, switch) as independent variables. Both main effects were significant, *F*_(2, 34)_ = 78.51, *MSe* = 13,422.07, η_*p*_^2^ = 0.82, *p* < 0.001 for Condition, and *F*_(1, 17)_ = 109.19, *MSe* = 7,323.94, η_*p*_^2^ = 0.86, *p* < 0.001 for Gating. The two-way interaction was also significant, *F*_(2, 34)_ = 32.41, *MSe* = 7,636.02, η_*p*_^2^ = 0.66, *p* < 0.001 (see Figure 2). The interaction is driven by marked gate switching effects in the ignore condition (reflecting gate closing), *F*_(1, 17)_ = 89.07, *MSe* = 8,903.23, $\eta_{p}^{2}$ = 0.84, *p* < 0.001, and the attend-task-switch condition (reflecting gate opening), *F*_(1, 17)_ = 55.09, *MSe* = 9,064.15, η_*p*_^2^ = 0.76, *p* < 0.001, but not in the attend-task-switch condition, *F*_(1, 17)_ = 0.50, *MSe* = 4,628.60, η_*p*_^2^ = 0.03, *p* = 0.49. In other words, the effect of gating disappeared when a task switching took place.

#### PE

A parallel ANOVA was conducted on the PE data. Only the main effect of Condition was significant, *F*_(2, 34)_ = 18.62, *MSe* = 0.0004, η_*p*_^2^ = 0.52, *p* < 0.001. A higher error rate was observed in the ignore condition compared to attend-task-repetition, *F*_(1, 17)_ = 9.28, *MSe* = 0.0002, η_*p*_^2^ = 0.35, *p* = 0.007, presumably reflecting the execution of the incorrect task in trials where the cue location was incompatible with the current task. In addition, accuracy in the attend-task-switch condition was lower than in the other conditions, *F*_(1, 17)_ = 21.03, *MSe* = 0.0006, η_*p*_^2^ = 0.55, *p* < 0.001. The main effect of Gating was non-significant, *F*_(1, 17)_ = 1.73, *MSe* = 0.0002, η_*p*_^2^ = 0.09*, p* = 0.21, and so was the interaction, *F*_(2, 34)_ = 1.12, *MSe* = 0.0004, η_*p*_^2^ = 0.06, *p* = 0.34.

In sum, the RT data showed an under-additive interaction was observed between gate opening and task-switching, so that the effect of gate opening was absent in the attend-switch condition. Specifically, the RT difference between *attend-repeat* and *attend-switch* in the gate repetition condition reflects the standard task-switching cost observed in typical task-switching experiments. No extra time was required to open the gate in the *attend-switch* condition, but gate opening without a task switch did involve a cost. As suggested above, this pattern might suggest that gate opening is part of task switching cost. That is, in typical task switching experiment, the gate opens in switch trials and closes thereafter (or, in a probabilistic manner throughout a sequence of repetition trials; c.f. Kessler and Oberauer, 2014). Accordingly, it should be re-opened as part of updating WM with a new task-set.

## Experiment 2

The goal of Experiment 2 was to replicate the results of Experiment 1, with one notable change. In the previous experiment, the location of the digit inside the frame served as a task cue. This procedure confounded task switching with the need to perform a saccade from one target location to another. In the present experiment, the target was always presented in the center of the frame, and the task was cued by asterisks that were presented either above and below the digit or on its sides.

### Method

#### Participants

Twenty-nine students from Ben-Gurion University of the Negev participated in the study in return for course credit or monetary compensation. All the participants were right handed, and reported having no neuropsychological deficits or learning disabilities. One participant was removed from the analysis due to reporting using her fingers to keep track of the relevant task.

#### Procedure

The procedure of Experiment 1 was with the following changes. First, instead of cuing the task by the location within the frame, we used asterisks on each side of the digit (e.g., ^*^7^*^) to cue the parity task, and asterisks above and below the digit to cue the magnitude task. This way, changing the task cue was not confounded with a need to perform a saccade to a different location. The experiment was composed of 8 blocks of 32 trials, in which the conditions were selected at random with equal probabilities. The first block was a practice block and was later removed from the analysis.

### Results

#### RT

**Figure 3:**
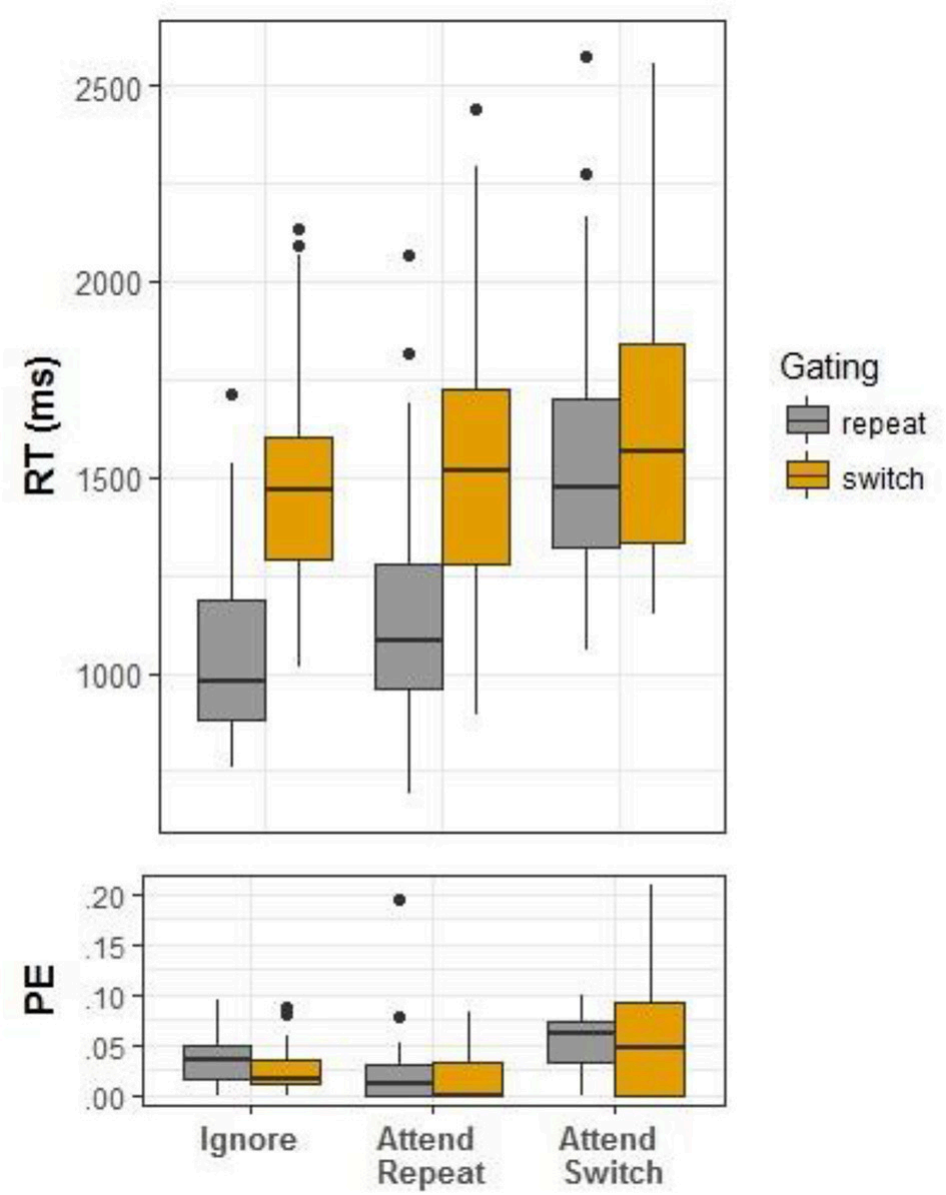
Box plots for reaction time (RT) and error proportions (PE) in Experiment 2.

Trial exclusion criteria were as in Experiment 1 (the proportion of trimmed trials was 0.0006 and 0.007%, respectively, in the two stages of RT trimming). An ANOVA was conducted with Condition and Gating as independent variables. The main effect of Condition was significant, *F*_(2, 54)_ = 41.06, *MSe* = 36,136.81, η_*p*_^2^ = 0.60, *p* < 0.001, as well as the main effect of Gating, *F*_(1, 27)_ = 75.59, *MSe* = 46,673.96, η_*p*_^2^ = 0.074, *p* < 0.001. The two-way interaction was also significant, *F*_(2, 54)_ = 48.05, *MSe* = 13,433.86, η_*p*_^2^ = 0.64, *p* < 0.001 (see Figure 3). As in Experiment 1, the effect of Gating was significant in the ignore condition, *F*_(1, 27)_ = 87.00, *MSe* = 29,986.45, η_*p*_^2^ = 0.76, *p* < 0.001, and the attend-task-repetition condition, *F*_(1, 27)_ = 78.87, *MSe* = 27,700.58, η_*p*_^2^ = 0.74, *p* < 0.01, but not in attend-task-switch, *F*_(1, 27)_ = 1.62, *MSe* = 15,854.64, η_*p*_^2^ = 0.06, *p* = 0.21.

#### PE

As in Experiment 1, the only the main effect of Condition was significant, *F*_(2, 54)_ = 24.68, *MSe* = 0.0008, η_*p*_^2^ = 0.48, *p* < 0.001. Again, more errors were observed in the ignore condition compared to attend-task-repetition, *F*_(1, 27)_ = 5.13, *MSe* = 0.0006, η_*p*_^2^ = 0.16, *p* = 0.032. Also, the attend-task-switch condition was more error-prone compared to the two task-repetition conditions, *F*_(1, 27)_ = 37.10, *MSe* = 0.0010, η_*p*_^2^ = 0.58, *p* < 0.001. The main effect of Gating was non-significant, *F*_(1, 27)_ = 1.05, *MSe* = 0.0011, η_*p*_^2^ = 0.04, *p* = 0.32, and so was the interaction, *F*_(2, 54)_ = 0.56, *MSe* = 0.0010, η_*p*_^2^ = 0.02, *p* = 0.57.

In sum, this pattern of results replicated the finding of Experiment 1.

## General discussion

The present work married the reference-back, developed within the context of declarative WM, with the task-switching paradigm, in order to investigate the relationship between WM gating and task-switching. In two experiments, the cost of gate opening, namely moving from an *ignore* condition (in which WM is in a “maintenance mode,” holding the previously-relevant task-set intact) to an *attend* condition (“updating mode”), was eliminated when a task-switch took place. First, the finding of a gate opening cost in procedural WM provides additional evidence for similar processes that act upon declarative and procedural memoranda (Oberauer et al., 2013). Furthermore, the under-additive interaction suggests that gate opening and task-switching operate in parallel.

Parallel processing, as observed here, implied that gate opening and task-switching are two distinct processes. While previous work (Kessler and Oberauer, 2014, 2015; Rac-Lubashevsky and Kessler, 2016a,b) consistently observed a gate switching cost, one possible interpretation that was not previously ruled out is that this cost does not reflect gating, but simply switching between two tasks. Applied to the reference-back paradigm, this interpretation holds that the reference and comparison conditions are mapped to two task-sets, one requiring WM updating and one not. Thus, switching between them gives rise to a cost, that has nothing to do with changing the state of the gate to WM, but only with switching between tasks. The present results do not support such an interpretation. If switching between trial-types is a special case of task-switching, then the two switch costs would have been additive or over-additive. The under-additive interaction observed here supports the notion that switching between trial-types is not merely task-switching, but rather reflects a different process, namely gate-switching.

The present findings establish gate-opening as a separate process than task-switching. Given this interpretation, the relationship between the two processes, and more specifically the involvement of gate opening in task switching, needs to be examined. The term “gating” is used in two different meaning (see McNab and Dolan, 2014). The first, which is implicated in the reference-back paradigm, is when relevant and relevant information alternate in time, and the relevant information needs to be protected (“gated”) from being overridden by subsequent distraction. A different usage of gating is as a metaphor for selective attention that operates on displays that include both targets and distractors, or—in task switching scenarios—on multivalent stimuli that serve as retrieval cues for both the relevant and irrelevant tasks. In the latter case, the function of gating is not to protect from irrelevant information that is presented perceptually, but rather from competing task-sets. Specifically, phenomena such as the task-rule congruency effect (Meiran and Kessler, 2008; Kessler and Meiran, 2010) and the automatic retrieval of stimulus-task associations (Waszak et al., 2003) suggest that the irrelevant task representation competes with the relevant one. Several control mechanisms were identified as counteracting forces for this interference, including backward inhibition (Mayr and Keele, 2000), competitor rule suppression (Meiran et al., 2010), and functional decay of the relevant task-set (Altmann and Gray, 2002).

Dreisbach and colleagues have demonstrated that the representation of categorical task rules, rather than individual S-R rules, shields against interference from irrelevant information (see Dreisbach, 2012, for review). Importantly for our discussion, this shielding needs to be relaxed when switching between task-sets, leading to increased interference in switch trials (Dreisbach and Wenke, 2011). Based on this idea, Dreisbach (2012) suggested that “the switch costs in the task-switching paradigm can be explained at least in part by the temporary relaxation of task shielding” (p. 229). The findings of the present study may provide an initial empirical support for this idea. Specifically, the under-additivity that was observed between the two processes is typically interpreted as independence. However, another interpretation would be that gate opening is one of the sub-processes that compose task switching, and that task switching cost includes the duration of gate opening. Under this account, gate opening can take place either separately, or as part of task switching. In the latter case, its associated cost is “absorbed” by task switching cost. This is because task switching cost is longer, since it also includes additional processes. More empirical work is needed in order to distinguish the two interpretations of under-additivity.

While the present work provided evidence for gate opening in task switching, the present paradigm does not enable to examine the possible association between task switching and gate closing. This is because moving from an *attend* trial to an *ignore* trial, namely gate closing, always involves a task repetition. Few possibilities are conceivable. First, it could be that gate closing occurs in switch trials, following response selection, in a manner that also contributes to task switching cost. A second possibility is that the gate closes immediately after the response in task-switch trials, and hence does not contribute to switching cost. A third option is that the gate does not necessarily close immediately, but the probability of closing increases throughout a sequence of task repetition trials (see Kessler and Oberauer, 2014, for a similar idea). Such a mechanism can balance the cost of gate closing with the need to shield the present goal. Future work is needed in order to better understand this issue.

## Author contributions

YK conducted the study, analyzed the data and wrote the paper.

### Conflict of interest statement

The author declares that the research was conducted in the absence of any commercial or financial relationships that could be construed as a potential conflict of interest.

## Appendix

**Table A1:** Means and SDs (in parentheses) for the RT and PE data.

**Condition:**	**Ignore**		**Attend-repeat**		**Attend-switch**	
**Gating:**	**Repeat**	**Switch (closing)**	**Repeat**	**Switch (opening)**	**Repeat**	**Switch (opening)**
**RT**						
**Exp. 1**	908 (142)	1,205 (238)	913 (172)	1,149 (205)	1,347 (211)	1,331 (186)
**Exp. 2**	1,061 (232)	1,494 (319)	1,155 (316)	1,550 (385)	1,568 (348)	1,611 (332)
**PE**						
**Exp. 1**	0.027 (0.017)	0.024 (0.016)	0.021 (0.015)	0.011 (0.011)	0.043 (0.029)	0.046 (0.032)
**Exp. 2**	0.035 (0.024)	0.026 (0.024)	0.024 (0.040)	0.016 (0.022)	0.055 (0.028)	0.057 (0.057)
